# Combining Bioorthogonal Chemistry with Fluorescent Silica Nanoparticles for the Ultrasensitive Detection of the HIV-1 p24 Antigen

**DOI:** 10.1021/acsomega.3c06136

**Published:** 2024-03-12

**Authors:** Tianwei Jia, Varma Saikam, Ying Luo, Xiaolin Sheng, Jieqiong Fang, Mukesh Kumar, Suri S. Iyer

**Affiliations:** † 788 Petit Science Center, Department of Chemistry, Center for Diagnostics and Therapeutics, Georgia State University, Atlanta, Georgia 30302, United States; ‡ 622 Petit Science Center, Department of Biology, Georgia State University, Atlanta, Georgia 30302, United States

## Abstract

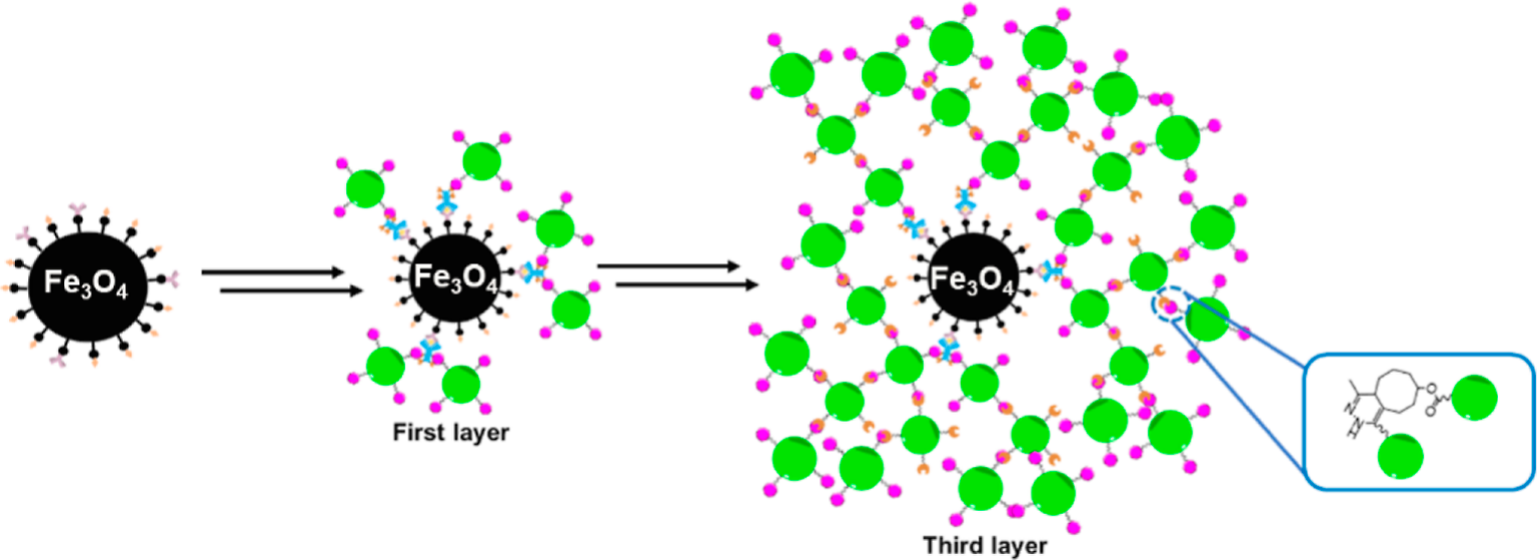

Early detection and viral concentration monitoring of human immunodeficiency virus in resource-poor settings are important to control disease spread and reduce mortality. Nucleic acid amplification tests are expensive for low-resource settings. Lateral flow antibody tests are not sensitive if testing is performed within 7–10 days, and these tests are not quantitative. We describe a signal enhancement technique based on fluorescent silica nanoparticles and bioorthogonal chemistries for the femtomolar detection of the HIV-1 p24 antigen. We developed a magnetic bead-based assay, wherein we used fluorescent-dye-encapsulated silica nanoparticles as reporters. The number of reporters was increased by using bioorthogonal chemistry to provide signal enhancement. The limit and range of detection of the sandwich immunoassay using alternating multiple layers for p24 in human serum were found to be 46 fg/mL (1.84 fM) and 46 fg/mL to 10 ng/mL, respectively. This simple assay was 217-fold higher in sensitivity compared to that of commercial enzyme-linked immunoassays (limit of detection of 10 pg/mL).

## Introduction

Human immunodeficiency virus (HIV) continues to be a major pathogen of importance since its identification in 1983.^1^ According to the 2020 WHO report, an estimated 38 million people are living with HIV with 0.7 million people dying from HIV-related causes.^2^ Early diagnosis plays a critical role in controlling disease spread and reducing mortality. HIV RNA can be detected by nucleic acid amplification tests (NAATs) approximately 7–10 days after infection; however, NAATs are expensive for use in resource-poor areas in endemic regions.^3^ The surrogate biomarker p24 antigen, a well-conserved protein with 2000–3000 copies in a single virion, can be detected by fourth-generation point-of-care (POC) lateral flow immunoassays approximately 15 days after infection.^4−6^ Despite these major advances, ultrasensitive assays to detect and monitor viral load in low-resource settings are needed. Ultrasensitive assays could be used for the (i) early detection of HIV in newborns born to HIV+ mothers. Since HIV can be transmitted via mother’s milk, it is quite conceivable that HIV– infants could become HIV+ if milk from an HIV+ mother is given to the infant.^7^ (ii) Virus concentration monitoring for HIV+ individuals at home or in low-resource settings since most physicians recommend changing the medication if there is a viral rebound to >1000 virus particles/mL (or 4 fM or 0.1 pg/mL of p24) of blood.^8,9^ Reports of ultrasensitive laboratory-based assays to detect femtomolar concentrations of p24 have been published.^10−16^ Commercial lateral flow assays, although inexpensive, are limited in scope for early detection or virus concentration monitoring as they cannot detect <1000 virus particles/mL. As a first step toward the development of ultrasensitive POC assays for early detection and viral monitoring, we developed assays to meet the desired femtomolar limits of detection (LODs) for p24 using dye-encapsulated fluorescent silica nanoparticles and bioorthogonal chemistries.

**Figure 1:**
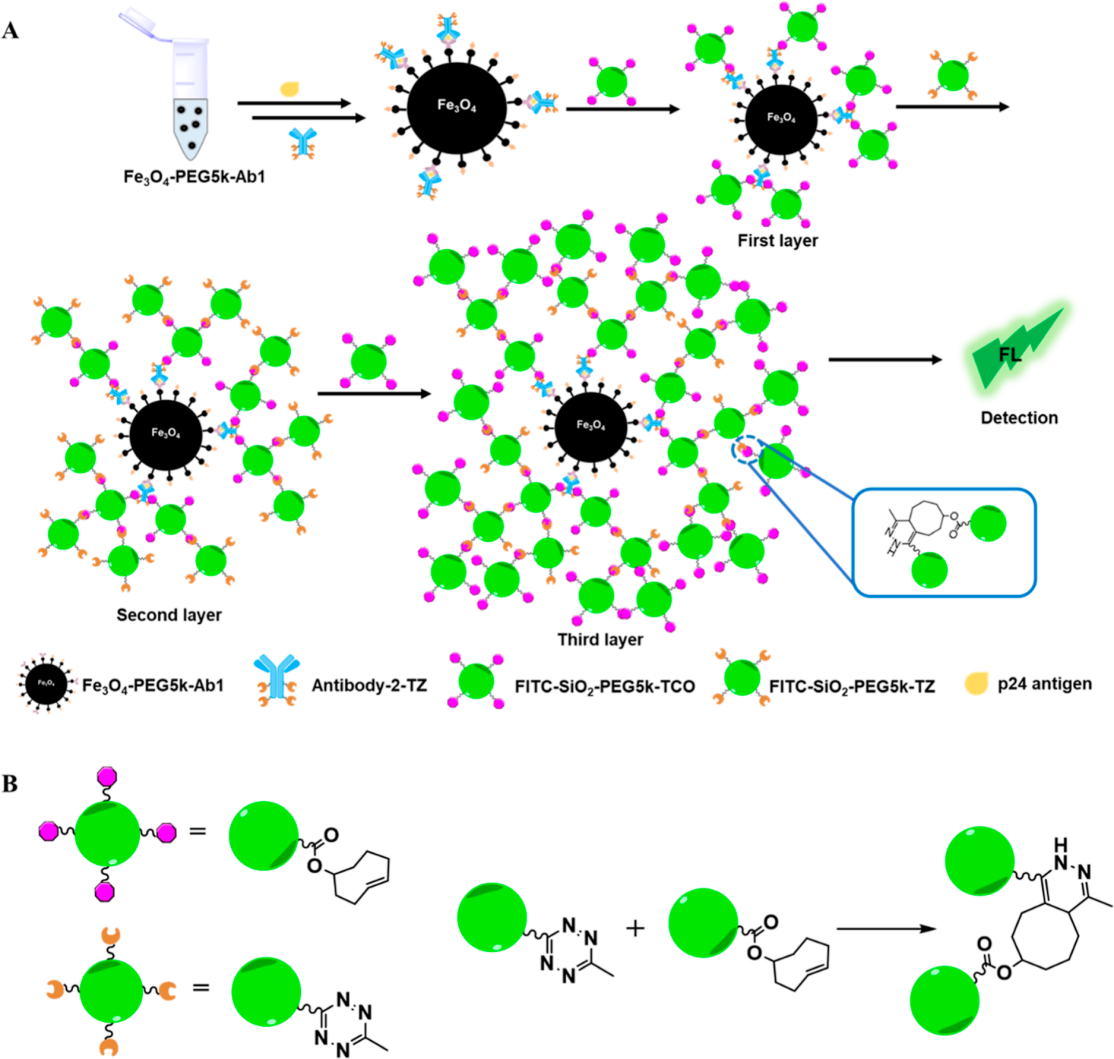
(A) Schematic representation of the multiple layers used to amplify the signal for ultrasensitive detection of p24. (B) Reaction schemes of FITC–SiO_2_–PEG–TCO and FITC–SiO_2_–PEG–TZ.

Our strategy was to develop a magnetic bead-based sandwich immunoassay using dye-encapsulated fluorescent silica nanoparticles and bioorthogonal chemistries to enhance the signal and lower the LOD for p24 (Figure 1). Fluorescent encapsulated silica nanoparticles have attracted strong interest in various applications such as bioimaging,^17−19^ biosensors,^20,21^ and diagnostics.^22,23^ These nanoparticles offer unique characteristics: (1) excitation and emission of light are favorable due to the silica matrix’s optical transparency;^24^ (2) a single nanoparticle has thousands of individual dye molecules that are caged, and therefore, there is minimal photobleaching, resulting in increased signal intensity compared to that of a single dye molecule;^25^ and (3) the silica matrix is photochemically inert and resistant to pH and temperature, and the large surface area can be easily functionalized for different applications.^26^ To improve the signal, we used a layer-by-layer approach. One of the major requirements for using a layer-by-layer approach is that the approach must be highly specific in ex vivo biological media, including blood, serum, sweat, urine, and tears. To this end, we relied on inverse electron demand Diels–Alder coupling reactions based on the high affinity and faster reaction times (∼1 to 10^6^ M^–1^ s^–1^) between 1,2,4,5-tetrazine (TZ) and *trans*-cyclooctene (TCO) without additional catalysts.^27^ TZ and TCO have been demonstrated to show bioorthogonality toward each other, making them ideal for our amplification assays.

The overall assay procedure is shown in Figure 1. First, magnetic beads were functionalized with anti-p24 antibodies. We used magnetic beads because they are relatively easy to separate from the solution using a magnet. Next, known concentrations of antigen p24 were added and washed to remove the unbound antigen. The secondary antibody, which has been modified with TZ, is added to the beads. This leads to a “sandwich” configuration, where the antigen is sandwiched between the two antibodies. After separation using a magnet and a wash step, fluorescent silica nanoparticles modified with TCO (FITC–SiO_2_–PEG5k–TCO) are added to the solution. The TCO reacts rapidly with the TZ present on the secondary antibody to yield the first layer. The first layer of FITC–SiO_2_–PEG5k–TCO provides a fluorescent signal, and importantly, it has a significant amount of unreacted TCOs. To develop the second layer, FITC–SiO_2_–PEG5k–TZ is added to the solution. The TZ present on FITC–SiO_2_–PEG5k–TZ reacts with the unreacted TCOs to form a second layer. Since there is unreacted TZ present in the second layer, addition of FITC–SiO_2_–PEG5k–TCO nanoparticles results in a third layer. In short, fluorescent nanoparticles with either TCO or TZ on the surface can be used to improve the signal in a layer-by-layer approach. Taken together, the assay increases the number of fluorescent dyes in a single capture event because (1) compared with a dye molecule, a fluorescent silica nanoparticle contains a large number of dye molecules, and (2) multiple layers of fluorescent nanoparticles by virtue of the biorthogonal chemistries enhance the total number of reporter molecules.^28^ For example, if a silica nanoparticle contains 1000 dye molecules and each layer adds a thousand silica nanoparticles, the amplification would be 1000 × 1000 or a million-fold compared to that of a single dye molecule. In comparison with a standard laboratory-based ELISA, there are no enzyme-linked antibodies or substrates required for signal amplification in this assay.

## Experimental Section

### Materials and Equipment

Tetraethyl orthosilicate (TEOS), 3-aminopropyl triethoxysilane (APTES), *N*-hydroxysuccinimide (NHS), and fluorescein isothiocyanate (FITC, isomer I) were purchased from Sigma-Aldrich. NH_4_OH (ammonium hydroxide, 28–30%) was purchased from ARISTAR ACS, VWR Chemicals BDH. The mouse anti-HIV-1 p24 paired antibody and recombinant HIV-1 p24 protein were purchased from Prospec Protein Specialists, USA. SuperMag carboxyl beads (200 nm) were purchased from Ocean NanoTech. Amine–PEG–valeric acid (NH_2_–PEG5k–COOH), PEG-bis–CH_2_COOH (HOOC–PEG5k–COOH), TCO–PEG6–amine HCl salt, and methyltetrazine–PEG4–amine HCl salt were purchased from BroadPharm. *N*-Hydroxysulfosuccinimide sodium salt (Sulfo-NHS) was purchased from TCI America. Human serum type AB (male) from male AB plasma was purchased from Sigma-Aldrich. Ultrapure water obtained from a Millipore water purification system (18.2 MΩ cm^–1^, Milli-Q, Merck Millipore, Darmstadt, Germany) was used in all experiments. Fluorescence intensity was detected by a Gen 5 Synergy LX Multimode reader, and a green filter was used in the assays (BioTek Instruments, Inc.). Transmission electron microscopy (TEM) images were generated by using a Talos L120C instrument. Bruker Daltonics ultrafleXtreme MALD TOF-TOF was used for MALDI-TOF mass spectrometry.

### Synthesis of FITC–SiO_2_–OH

FITC–SiO_2_–OH was prepared according to the reported procedures with modifications.^29^ In a 25 mL round-bottomed flask capped with a rubber septum, 5 mL of absolute ethanol was mixed with 10 mg of FITC and 20 μL of APTES under a nitrogen atmosphere. The mixture was stirred for 15 h at 25 °C to obtain the FITC–APTES adduct. 20 mL of ethanol, 2.0 mL of TEOS, 0.65 mL of NH_4_OH, and 1.3 mL of Milli-Q-grade water were added. The reaction proceeded for 24 h. The yellow dispersion was washed with absolute ethanol ten times (30 mL) through cycles of centrifugation (7900*g*, 12 min)/sonication/redispersion. Finally, the material was redispersed in 10 mL of absolute ethanol.

### Synthesis of FITC–SiO_2_–NH_2_

The surface modification of FITC–SiO_2_–OH with APTES was performed in an ethanol solution at 95 °C. A 200 μL portion of APTES was added to 60 mg of FITC–SiO_2_–OH in 15 mL of ethanol. The mixture was stirred for 24 h. FITC–SiO_2_–NH_2_ was separated from the mixture by centrifugation (16,128*g*, 10 min) and washed with ethanol three times. The ethanol was removed, and the material was dried in vacuo for 2 h.

### Synthesis of FITC–SiO_2_–PEG5k–COOH

HOOC–PEG5k–COOH (55 mg, 11 μmol) was dissolved in 2 mL of DMF. EDC–HCl (1.9 mg, 10 μmol, dissolved in DMF) and NHS (1.15 mg, 10 μmol, dissolved in DMF) were added. The mixture was stirred at room temperature (rt) for 30 min. 30 mg of FITC–SiO_2_–NH_2_ suspended in 1.0 mL of DMF was added and stirred for 24 h. The obtained nanoparticles were separated from the mixture by centrifugation (16,128*g*, 10 min) and washed with ethanol three times. The ethanol was removed, and the material was dried in vacuo for 2 h.

### Fabrication of 100 nm FITC–SiO_2_–PEG5k–TCO Nanoparticles

5 mg of FITC–SiO_2_–PEG5k–COOH was resuspended in 1 mL of DMF. EDC–HCl (1.9 mg, dissolved in 200 μL DMF) and NHS (1.15 mg, dissolved in 200 μL DMF) were added to the solution. The mixture was stirred at rt for 1 h. TCO–PEG6–NH_2_ (2 mg, 4 μmol) dissolved in 200 μL of DMF was added to the mixture and stirred for 24 h. The resulting nanoparticles were separated by centrifugation (7400*g*, 10 min), washed with 1 mL of ethanol (three times), and 1 mL of PBS (three times). The final FITC–SiO_2_–PEG5k–TCO nanoparticles were resuspended in 1 mL of PBS (5 mg/mL). The resulting stock solution was stored at 4 °C for further experimentation.

### Fabrication of 100 nm FITC–SiO_2_–PEG5k–TZ Nanoparticles

These nanoparticles were fabricated in a manner similar to the fabrication of FITC–SiO_2_–PEG5k–TCO using TZ–PEG4–NH_2_ instead of TCO–PEG4–NH_2._

### Synthesis of Fe_3_O_4_–PEG5k–COOH

500 μg of carboxyl Fe_3_O_4_ nanoparticles was washed with pH 6 buffer (three times) and resuspended in 300 μL of PBS buffer (pH 6). 100 μL of EDC–HCl (100 μL, 100 mg/mL, dissolved in PBS buffer, pH 6) and Sulfo-NHS (100 mg/mL, dissolved in PBS buffer, pH 6) was added to the beads in a 1.5 mL microcentrifuge tube and incubated at rt for 1 h. After incubation, the activated nanoparticles were washed with PBS buffer (pH 7.4) once and resuspended in 400 μL of PBS buffer (pH 7.4). 2 mg of NH_2_–PEG5k–COOH was dissolved in 100 μL of PBS buffer (pH 8). A 50 μL aliquot of NH_2_–PEG5k–COOH was added to the microcentrifuge tube. After 5 min, 50 μL of NH_2_–PEG5k–COOH was added to the mixture and incubated at rt for 24 h. Fe_3_O_4_–PEG5k–COOH was separated using a magnet, washed three times with 500 μL of PBS, and stored in 500 μL of PBS at 4 °C.

### Fabrication of Fe_3_O_4_–PEG5k–Antibody (Fe_3_O_4_–PEG5k–Ab) Magnetic Beads

480 μg of Fe_3_O_4_–PEG5k–COOH magnetic beads was dispersed in 300 μL of PBS buffer (pH 6). 100 μL of EDC–HCl (100 μL, 100 mg/mL, dissolved in PBS buffer, pH 6) and Sulfo-NHS (100 mg/mL, dissolved in PBS buffer, pH 6) was added to the magnetic beads in a 1.5 mL microcentrifuge tube and incubated at rt for 1 h. The activated particles were washed with PBS buffer (pH 5.5) and resuspended in 450 μL of PBS buffer (pH 5.5). 50 μL of 50 μg of monoclonal antibody was added to the microcentrifuge tube and incubated for 15 min. 25 μL of PBS buffer (pH 11.6) was added to the mixture dropwise to change the reaction solution to pH 8. The reaction mixture was stirred for 2.5 h. The magnetic beads were separated using a magnet and washed with 0.5 mL of PBS three times. The unreacted carboxylic group was blocked with a PBS wash buffer (0.1% BSA, 0.05% Tween 20, pH 7.4) at 25 °C for 0.5 h. The final Fe_3_O_4_–PEG5K–antibody magnetic beads were separated, washed three times with 0.5 mL PBS, and stored in 470 μL PBS at 4 °C.

### Preparation of the Tetrazine-Modified Antibody (Ab2–TZ)

100 μg of p24 antibody was dispersed in 300 μL of PBS buffer (pH 7.4). A solution of 100 μL of EDC–HCl (10 mg/mL in PBS buffer, pH 7.4) and 100 μL of Sulfo-NHS (10 mg/mL in PBS buffer, pH 7.4) was added to the p24 antibodies in a 1.5 mL microcentrifuge tube and incubated at rt for 0.5 h. 1000 molar equiv of TZ–PEG4–NH_2_ (25 mM, 26.68 μL) was added to the solution. The reaction mixture was stirred for 5 h. Ab–TZ was purified by Nanosep 30K. The concentration of Ab2–TZ was identified using BCA assays and stored at −20 °C.

### Feasibility Studies

In a 0.2 mL PCR tube, Fe_3_O_4_–PEG5k–Ab (10 μL, 10 μg) was suspended in 30 μL of PBS buffer (0.1% BSA, 0.05% Tween 20, pH 7.4). The p24 antigen (10 μL, 50 pg/mL) was added and incubated for 30 min at rt. After separation using a magnet, the magnetic beads were washed two times with 50 μL of PBS wash buffer (0.1% BSA, 0.05% Tween 20, pH 7.4), and the magnetic beads were resuspended in 40 μL of PBS wash buffer. Ab2–TZ (10 μL, 1 μg) was added and incubated for 30 min. After separation using a magnet, the magnetic beads were resuspended in 40 μL of PBS buffer (0.1% BSA, 0.05% Tween 20, pH 7.4) and incubated with FITC–SiO_2_–PEG5k–TCO (10 μL, 50 μg) for 30 min. After separation using a magnet, the magnetic beads were washed with 50 μL of PBS buffer (0.1% BSA, 0.05% Tween 20, pH 7.4) three times. The final magnetic bead fluorescent nanoparticle complex (first layer) was resuspended in 50 μL of PBS wash buffer, and the fluorescence was recorded at a wavelength of 480/520 nm. Next, the first layer complex comprising the magnetic bead fluorescent nanoparticle complex was transferred to a 0.2 mL PCR tube, and 10 μL of 50 μg of FITC–SiO_2_–PEG5k–TZ was added. After 30 min of incubation, the resulting complex was separated using a magnet and washed with 50 μL of PBS buffer (0.1% BSA, 0.05% Tween 20) three times. The magnetic beads fluorescent nanoparticle complex (second layer) was resuspended in 50 μL of PBS wash buffer, and fluorescence was recorded at a wavelength of 480/520 nm. The third layer was formed in a manner similar to the formation of the second layer using FITC–SiO_2_–PEG5k–TCO, and fluorescence was recorded at a wavelength of 480/520 nm.

### Determining the Limit and Range of Detection in PBS and Serum Samples

To determine the sensitivity of the platform, different concentrations of p24 (10 μL, 0–50 ng/mL) in PBS were used. Fe_3_O_4_–PEG5k–Ab (5 μL, 5 μg), FITC–SiO_2_–PEG5k–TCO (10 μL, 50 μg), FITC–SiO_2_–PEG5k–TZ (10 μL, 50 μg), and Ab2–TZ (10 μL, 1 μg) were used for all analyses. The procedure was similar to the one described for the feasibility studies. For determining the limit and range of detection in human serum, different concentrations of p24 (10 μL, 0–50 ng/mL) were spiked in commercial human serum samples, and the same procedure was followed as described for the analysis of p24 in PBS.

Additional details of the fabrication of the dye-doped fluorescent nanoparticles, surface modification, zeta potentials, TEM images, and MALDI-TOF analysis of the antibody–TZ conjugates are given in the Supporting Information.

## Results and Discussion

**Figure 2:**
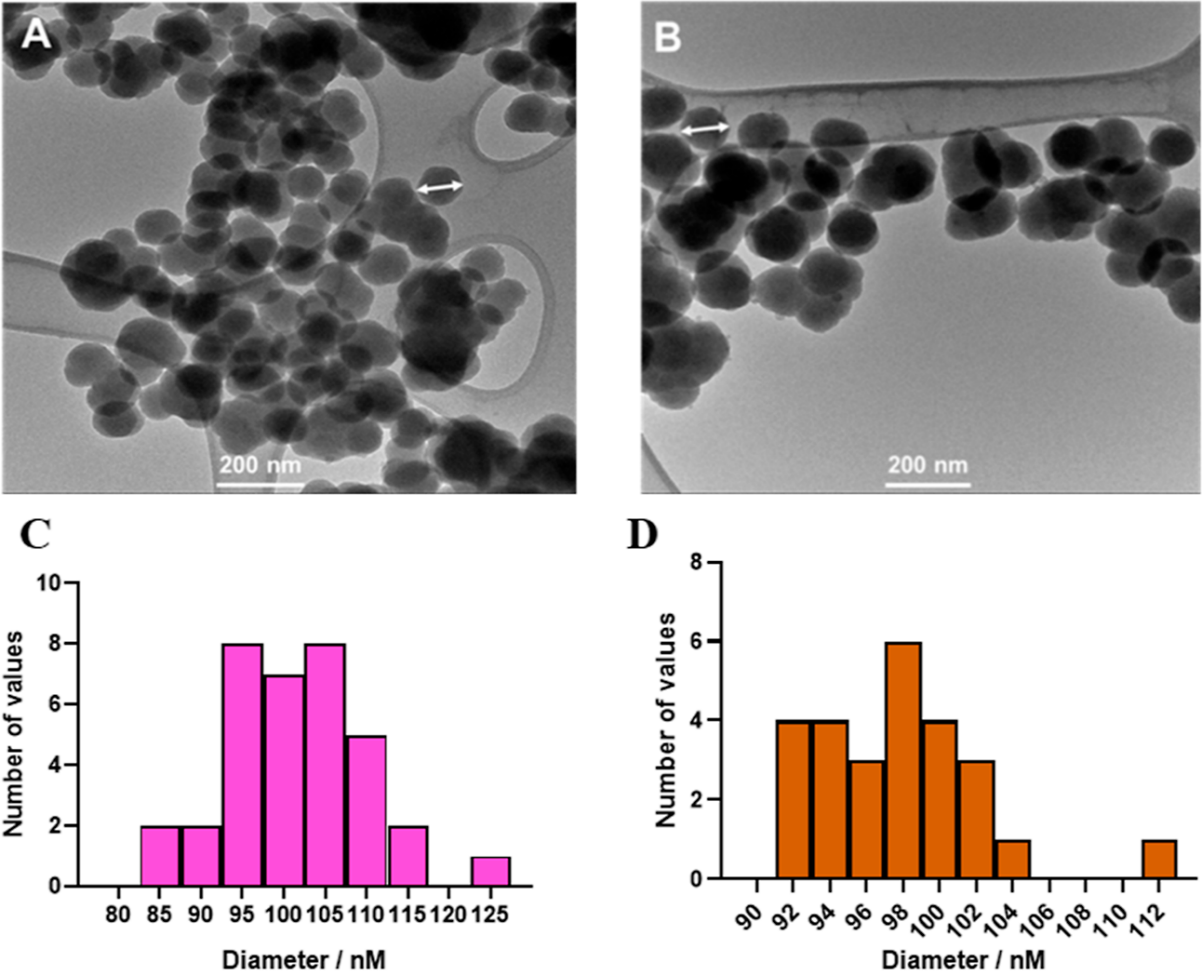
TEM images of fluorescent silica nanoparticles. (A) FITC–SiO_2_–PEG5k–TCO. (B) FITC–SiO_2_–PEG5k–TZ. Size distribution of (C) FITC–SiO_2_–PEG5k–TCO and (D) FITC–SiO_2_–PEG5k–TZ. The white arrows indicate the size of the fluorescent silica nanoparticles.

We generated all of the materials and characterized them extensively before performing the assays (Figures S1–S5). The materials were the antibody conjugated to the magnetic beads, TZ conjugated to the secondary antibody, and dye-doped fluorescent nanoparticles with either TZ or TCO on the surface. To generate Fe_3_O_4_–PEG5k–Ab magnetic beads, we relied on reported protocols with some modifications.^30,31^ We introduced a poly(ethylene glycol) (PEG5k, molecular weight of 5000 g/mol) spacer between the magnetic beads and the antibody to reduce nonspecific binding for all conjugated materials. The zeta potential of the starting material, Fe_3_O_4_–PEG5k–COOH, was determined to be −7.16 ± 0.76, indicating an overall negative charge on the magnetic bead. In contrast, the zeta potential of the product, Fe_3_O_4_–PEG5k–Ab, was determined to be 3.30 ± 1.37. This difference in the zeta potential clearly indicates that the antibody was conjugated to the magnetic bead (Figure S4A). Next, a tetrazine-modified antibody (Ab–TZ) was synthesized using standard acid-based chemistries and was characterized by MALDI-TOF analysis. The molecular weight of the antibody was determined to be 150,039 Da, and the molecular weight of Ab–TZ was 153,535 Da. Since the molecular weight of TZ is 363 Da, the antibody has an average of 10 TZ units (Figure S5). Finally, dye-doped fluorescent silica nanoparticles were generated and conjugated to either TCO or TZ on their surfaces. 100 nm dye-doped fluorescent nanoparticles were prepared using published reports, and the surface was modified with a polyethylene glycol spacer that was terminated with a carboxyl group.^29^ Next, either TCO or TZ was conjugated with fluorescent silica nanoparticles to yield FITC–SiO_2_–PEG5k–TCO or FITC–SiO_2_–PEG5k–TZ, respectively. The zeta potentials for FITC–SiO_2_–PEG5k–TCO and FITC–SiO_2_–PEG5k–TZ were −3.74 ± 0.67 and −2.52 ± 0.47, respectively (Figure S4B). Additionally, FITC–SiO_2_–PEG5k–TCO and FITC–SiO_2_–PEG5k–TZ were evaluated by dynamic light scattering (DLS) (Figure S3). The size of these fluorescent nanoparticles was 100 nm, as confirmed by TEM analysis (Figures 2 and S2).

**Figure 3:**
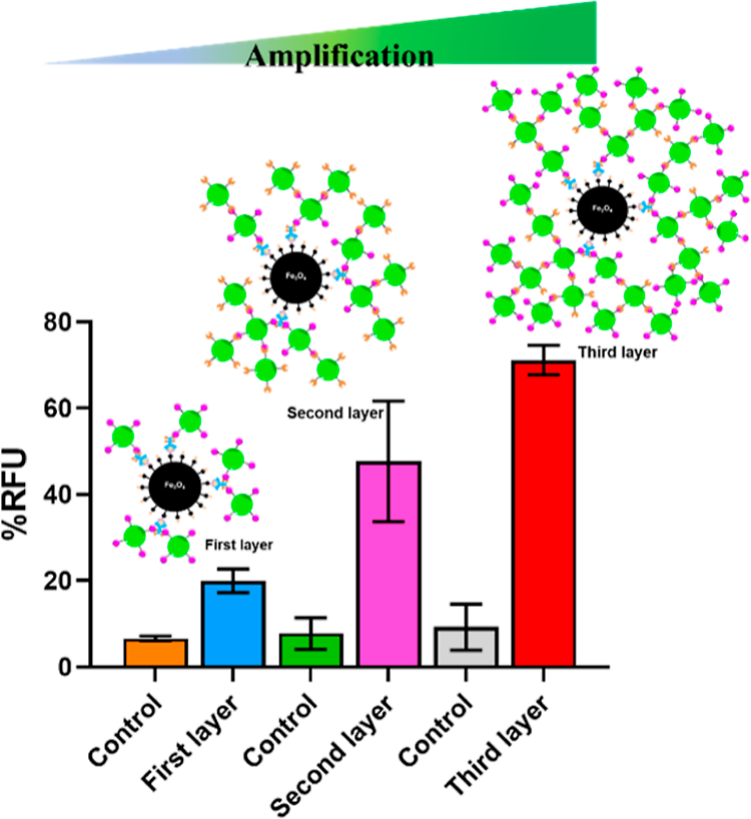
Demonstration of the signal amplification strategy using the HIV p24 antigen. The *y*-axis, % RFU, represents the % relative fluorescence intensity of the sample as a function of an internal control. Error bars indicate the standard deviations of three measurements performed on three different days.

With the materials in hand, we developed the assay. Fe_3_O_4_–PEG5k–Ab was introduced into samples containing the p24 antigen. After separation with a magnet, the magnetic particles were washed and resuspended in PBS buffer, and the anti-p24-TZ conjugate was added. After a brief incubation period, FITC–SiO_2_–PEG5k–TCO was added, separated with a magnet, and washed with PBS, and the fluorescence intensity was measured. This was the first layer, as shown in Figure 1. FITC–SiO_2_–PEG5k–TZ was added to this complex after resuspension to give it a second layer. The third layer was generated using FITC–SiO_2_–PEG5k–TCO. As seen in Figure 3, the signal of the first layer was higher than the control (no antigen added), indicating that FITC–SiO_2_–PEG5k–TCO conjugated to Ab–TZ via the TCO–TZ bioorthogonal reaction. The second layer showed increased signal intensity compared with that of the first layer, while the signal of the control group was almost unchanged. The results suggest that the unreacted TCO group reacts with the paired FITC–SiO_2_–PEG5k–TZ. The residual TZ on the second layer’s FITC–SiO_2_–PEG5k–TZ surface was used to amplify the signal by reacting with FITC–SiO_2_–PEG5k–TCO. The third-layer signal significantly increased compared to that of the control group. We observed a slight increase in the standard deviation of the control group, presumably due to nonspecific binding. Taken together, successive increases in fluorescent signal in each round indicated the feasibility of the sandwich immunoassay to amplify the signal and lower the LOD.

**Figure 4:**
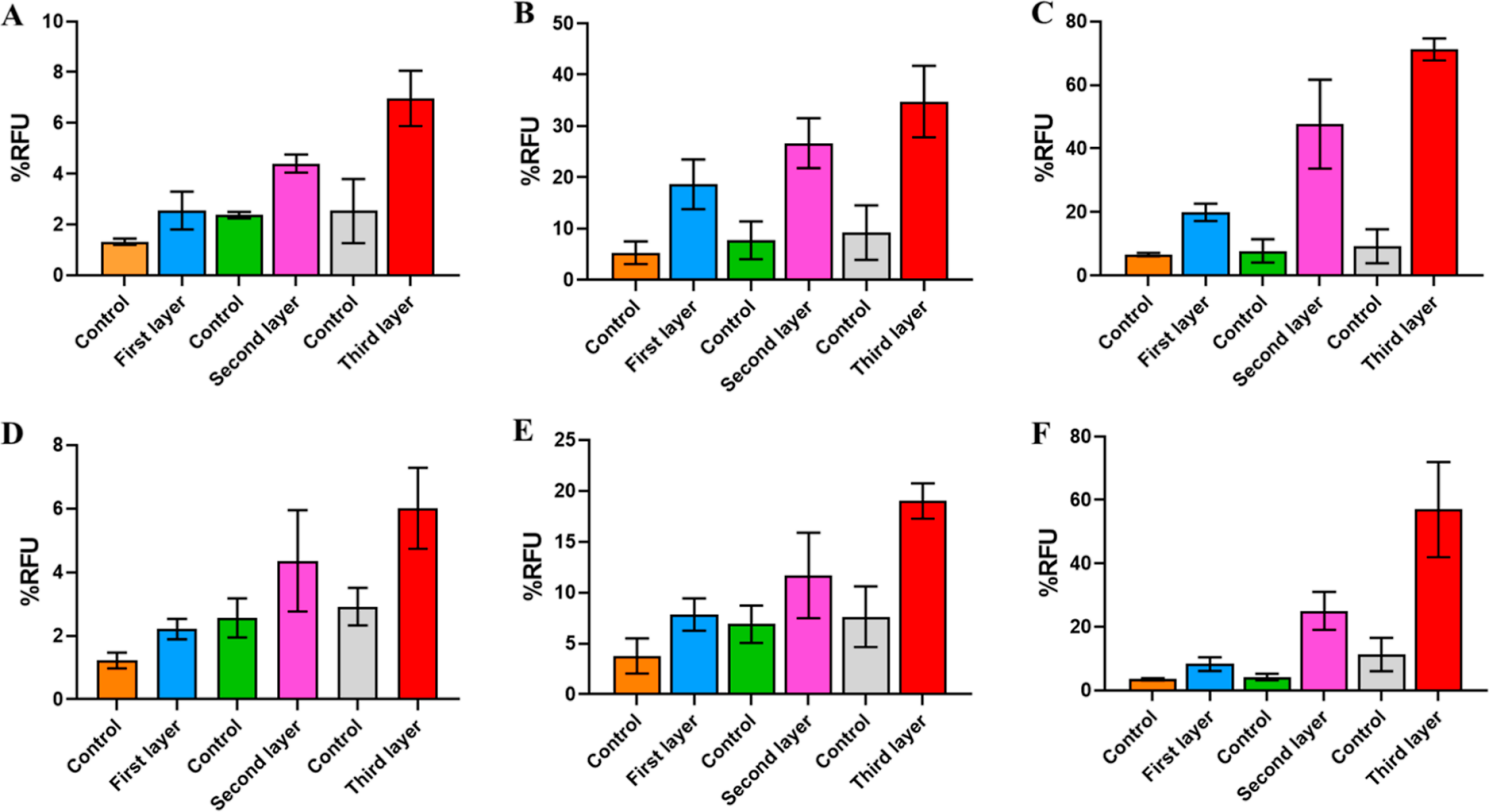
Optimization studies. (A–C) represent 10 μg of Fe_3_O_4_–PEG5k–Ab and (D–F) represent 5 μg of Fe_3_O_4_–PEG5k–Ab. (A,D) represent 5 μg of FITC–SiO_2_–PEG5k–TZ/TCO, (B,E) represent 25 μg of FITC–SiO_2_–PEG5k–TZ/TCO, and (C,F) represent 50 μg of FITC–SiO_2_–PEG5k–TZ/TCO. The *y*-axis, % RFU, represents the relative fluorescence intensity of the sample as a function of an internal control. Error bars indicate the standard deviations of three measurements performed on three different days.

Next, we optimized the system by varying the amount of Fe_3_O_4_–PEG5k–Ab magnetic beads and dye-doped fluorescent silica nanoparticles. The amount of fluorescent nanoparticles is important; higher amounts lead to nonspecific binding, whereas a lower amount is not sufficient for signal enhancement. We used 5 or 10 μg of the magnetic beads and 5, 25, and 50 μg of the fluorescent nanoparticles for the optimization studies. The data shown in Figure 4 show that the amount, 5 or 10 μg, of magnetic beads does not result in a major improvement in the results. However, when comparing 5, 25, or 50 μg of fluorescent nanoparticles, we find that using 50 μg results in a higher signal and minimal nonspecific binding. The signal-to-background ratio for the different conditions is summarized in Table 1. The best signal-to-background ratio is observed when we use 5 μg of Fe_3_O_4_–PEG5k–Ab and 50 μg of FITC–SiO_2_–PEG5k–TZ/TCO, and these amounts were used to determine the limit and range of detection.

**Table 1:** Signal-to-Background Ratio Using Different Amounts of Magnetic Beads and Nanoparticles

	10 μg of Fe_3_O_4_–PEG5k–Abl			5 μg of Fe_3_O_4_–PEG5k–Abl		
	5 μg of FITC–SiO_2_–PEG5k–TZ/TCO	25 μg of FITC–SiO_2_–PEG5k–TZ/TCO	50 μg of FITC–SiO_2_–PEG5k–TZ/TCO	5 μg of FITC–SiO_2_–PEG5k–TZ/TCO	25 μg of FITC–SiO_2_–PEG5k–TZ/TCO	50 μg of FITC–SiO_2_–PEG5k–TZ/TCO
first layer	1.91 ± 0.43	2.20 ± 0.99	3.00 ± 0.25	1.92 ± 0.73	2.30 ± 0.74	2.40 ± 0.84
second layer	1.86 ± 0.15	4.15 ± 2.25	6.06 ± 3.08	1.85 ± 1.05	1.67 ± 0.17	5.96 ± 1.12
third layer	3.62 ± 2.62	4.29 ± 1.43	4.90 ± 1.08	2.17 ± 0.92	2.79 ± 1.19	5.54 ± 2.23

**Figure 5:**
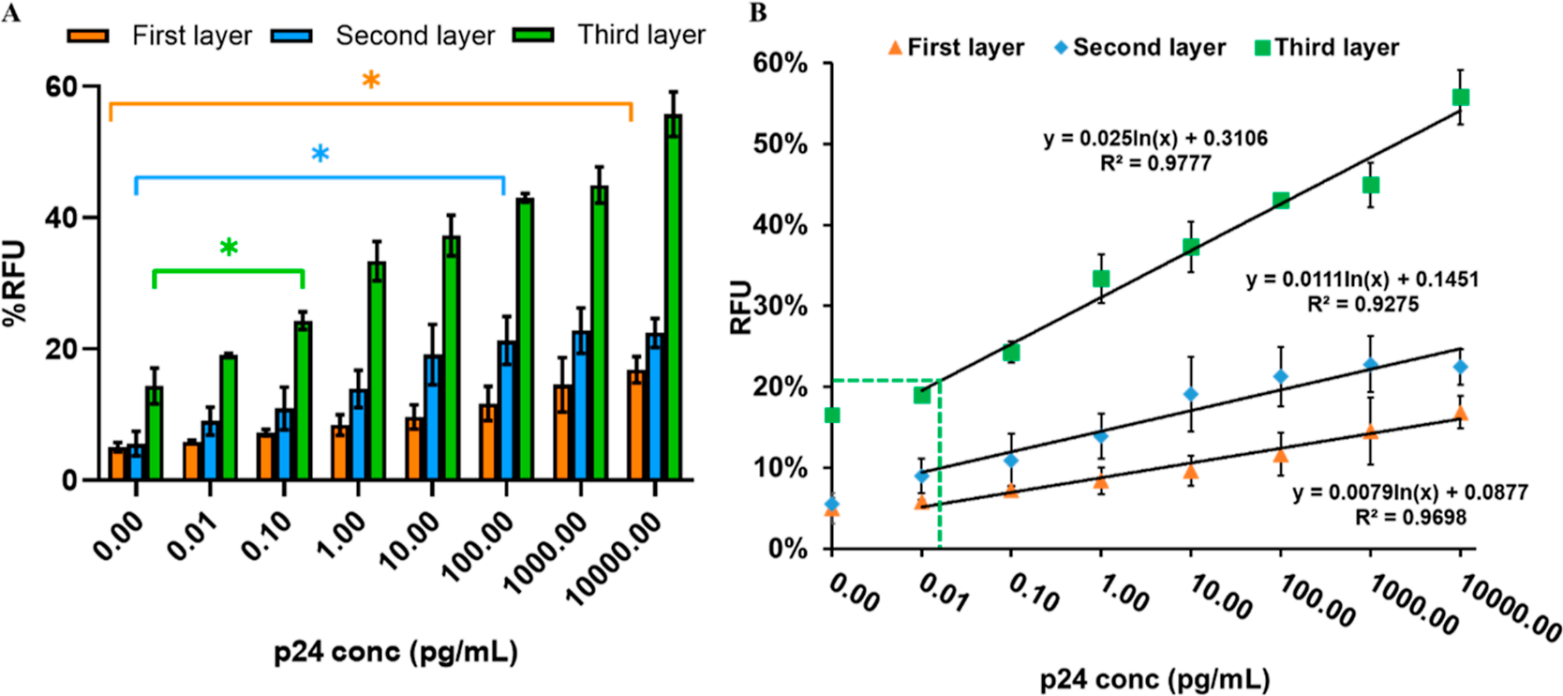
Signal response of the sandwich immunoassay using multiple layers. (A) Quantification analysis of the p24 antigen in PBS. (B) The linear relationship between the signal value and the different concentrations of the p24 antigen in PBS and the green dashed line indicate the LOD of the third layer. The *y*-axis, % RFU, is the percent relative fluorescence intensity of the sample as a function of an internal control. Error bars indicate the standard deviations of three measurements. (ns > 0.05, *p < 0.05.)

To determine the analytical sensitivity of the sandwich immunoassay, different concentrations of the recombinant p24 antigen (0–10 ng/mL or 0–400 pM) were used (Figure 5A). We found signal enhancement in all three layers for all tested concentrations. We could detect 10 ng/mL (400 pM) in comparison with the control group (absence of the p24 antigen, *p* < 0.05) for the first layer. For the second and third layers, we could detect 100 pg/mL (4 pM, *p* < 0.05) and 0.1 pg/mL (4 fM, *p* < 0.05), respectively. The second layer shows a 100-fold increase in sensitivity compared to that of the first layer, and the third layer shows a 1000-fold increase in sensitivity compared to that of the second layer. The increase in sensitivity for the third layer is 100,000-fold compared to that of the first layer. The linear relation equation could be fitted to *y* = 0.0576*x* + 0.3106, *R*^2^ = 0.9777, where *x* is the concentration of the p24 antigen and *y* is the % RFU in the third layer. The value of the LOD is calculated using the formula: LOD = mean blank value plus 3σ, where σ represents the value of the standard deviation of blank samples. According to the formula, the LOD was calculated to be 17 fg/mL (0.68 fM) in the third layer. The range of detection for the third layer was from 17 fg/mL to 10 ng/mL (Figure 5B). Our strategy exhibited 580-fold higher analytical sensitivity compared to that of conventional enzyme immunoassays, where the LOD is 10 pg/mL.^10,32^ Most importantly, it was gratifying to observe that this assay could reach our goal of femtomolar-level sensitivity for the p24 antigen.

**Figure 6:**
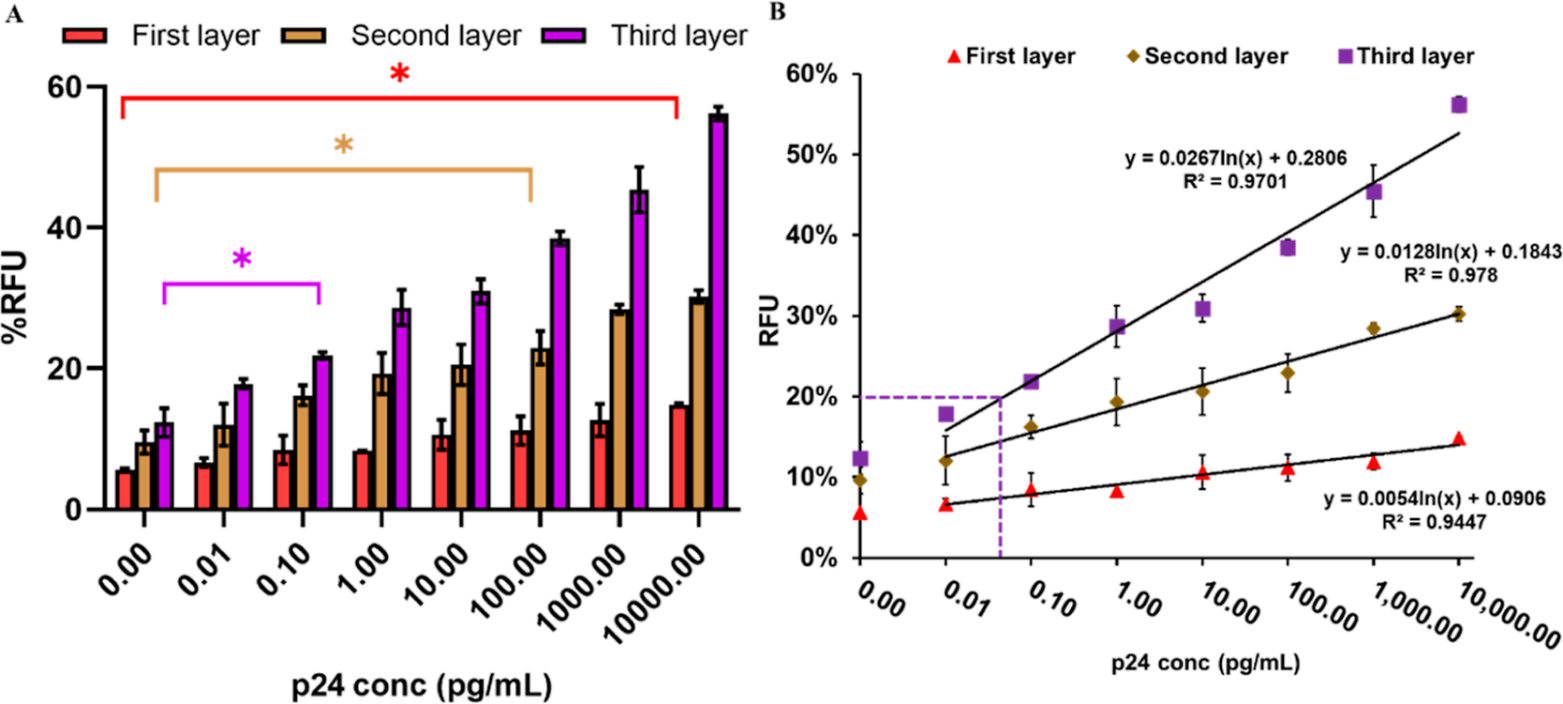
Signal response of the sandwich immunoassay alternating multiple layers signal amplification strategy. (A) Quantification analysis of the p24 antigen in human serum. (B) The linear relationship between the signal value and the different concentrations of the p24 antigen in human serum and the purple dashed line indicate the LOD of the third layer. The *y*-axis, % RFU, is the percent relative fluorescence intensity of the sample as a function of an internal control. Error bars indicate the standard deviations of three measurements. (ns > 0.05, *p < 0.05.)

Next, we tested the performance of this assay in human serum samples (Figure 6). Recombinant p24 was spiked in commercial human serum samples and subjected to the assays in a manner similar to the testing for p24 in PBS buffer. Similar to the detection of p24 in PBS buffer, an excellent detection was observed for p24 spiked in human serum. We could detect 0.1 pg/mL p24 for the third layer, while the first layer is 10 ng/mL and the second layer is 100 pg/mL, indicating enhanced sensitivity in each layer (*p* < 0.05). The third layer exhibits 1000-fold higher sensitivity than that of the second layer, and the second layer exhibits 100-fold higher sensitivity than that of the first layer. A linear relationship between the fluorescence signal and the concentration of the p24 antigen is found in the range of 10 fg/mL to 10 ng/mL (Figure 6B). The linear relation equation could be fitted to *y* = 0.0614*x* + 0.2806, *R*^2^ = 0.9701, where *x* is the concentration of the p24 antigen and *y* is the % RFU for the third layer. According to the formula, the LOD was calculated to be 46 fg/mL (1.84 fM) for the third layer. This approach yielded 217-fold higher analytical sensitivity compared to that of the conventional ELISA (10 pg/mL).^10,32^Table 2 compares this strategy with other methods involving colorimetric, fluorescence, and electrochemical assays that are used to detect the p24 antigen. In brief, our assay has a comparable LOD in the femtogram per microliter range to other methods.

**Table 2:** Comparison of the Analytical Sensitivity to Other HIV-1 p24 Biosensors^a^

detection methods	strategy	LOD	detection range	ref
fluorescence	streptavidin-conjugated AuNCs	5.0 pg/mL	up to 1000 pg/mL	(10)
fluorescence and visual	TdT, Cu NPs	0.025 fg/mL	0.025–1000 fg/mL	(11)
LFIA-naked eye	Pt NCs, CN/DAB	0.8 pg/mL	0.8–10,000 pg/mL	(12)
fluorescence	streptavidin labeled FSN	8.2 pg/mL	8.2–1000 pg/mL	(13)
fluorescence	β-sheets bind with Congo red	0.61 pg/mL (3F-based)	0.61–150 pg/mL	(14)
		2.44 pg/mL (2F-based)	2.44–150 pg/mL	
PEC	ALP-encapsulated liposomes	0.63 pg/mL	0.63–50,000 pg/mL	(15)
electrochemical	Fe_3_O_4_@SiO_2_Ab1/AuNPs/EV-p24 Ab2	0.5 pg/mL	0.5–10,000 pg/mL	(16)
fluorescence	layer-by-layersignal amplification	0.017 pg/mL (PBS)	0.017–10,000 pg/mL	this work
		0.046 pg/mL (serum)	0.046–10,000 pg/mL	

a AuNCs: gold nanoclusters. TdT: terminal deoxyribonucleotidyl transferase. Cu NPs: copper nanoparticles. LFIA: lateral flow immunoassays. Pt NCs: platinum core–shell nanocatalysts. CN/DAB: 4-chloro-1naphthol/3,3′-diaminobenzidine, tetrahydrochloride. FSN: fluorescent silver nanoparticle. PEC: photoelectrochemical. ALP: alkaline phosphatase. Fe_3_O_4_@SiO_2_: silicon dioxide-coated magnetic nanoparticles. AuNPs: gold nanoparticles. EV: a dextrin amine skeleton anchoring more than 100 molecules of HRP and 15 molecules of anti-IgG.

## Conclusions

We have developed a sandwich immunoassay using fluorescent silica nanoparticles and biorthogonal chemistries for the ultrasensitive detection of the HIV-1 p24 antigen. This strategy relies on a dual amplification system: (1) fluorescent silica nanoparticles containing a large number of dye molecules and (2) an increase in the number of fluorescent silica nanoparticles by alternating bioorthogonal chemistries in each round. The LOD of the sandwich immunoassay is 46 fg/mL (1.84 fM) in human serum, which exhibits higher analytical sensitivity when compared with that of the fourth-generation ELISA.^10,32,33^ The high sensitivity is required for early detection of the HIV-1 p24 antigen and allows patients to start antiretroviral treatment early. The high sensitivity also makes it possible for HIV+ individuals to determine if their virus concentration is >1000 particles/mL. According to the WHO guidelines, treatment failure of antiretroviral therapy is defined if the virus concentration is >1000 particles/mL for two consecutive tests and if the patient is compliant with the prescribed medication.^34^ If the concentration is >1000 particles/mL, healthcare professionals can change the medication. Finally, this assay has broad implications; disease monitoring can be achieved for a plethora of diseases by using different capture and detector antibodies or other recognition molecules.

## Supporting Information Available

Apples to apples comparison to demonstrate the LOD and range of detection of our assay versus a standard commercially available ELISA for p24, additional TEM images of fluorescent silica nanoparticles, DLS measurement, zeta potential of magnetic nanoparticles, and MALDI-TOF for Ab2–TZ (PDF)
