# Supplementary material for: Combining Bioorthogonal Chemistry with Fluorescent Silica Nanoparticles for the Ultrasensitive Detection of the HIV-1 p24 Antigen
Source: ACS Omega. 2024 Mar 12;9(12):14604–12. doi: 10.1021/acsomega.3c06136 (PMC10976350; doi:10.1021/acsomega.3c06136)
Supplement: Supplementary file 1 — ao3c06136_si_001.pdf [file ao3c06136_si_001.pdf]

Supporting Information

Combining Bioorthogonal Chemistry with Fluorescent Silica Nanoparticles for the Ultrasensitive Detection of HIV-1 p24 Antigen

Tianwei Jia,[1] Varma Saikam,[1] Ying Luo,[1] Xiaolin Sheng,[1] Jieqiong Fang,[1] Mukesh Kumar[2,*] and Suri S. Iyer[1, 3, *]

[1]788 Petit Science Center, Department of Chemistry, Center for Diagnostics and Therapeutics, Georgia State University,Atlanta, GA 30302, USA.

[2]622 Petit Science Center, Department of Biology, Georgia State University, Atlanta, GA 30302, USA. Email: mkumar8@gsu.edu

[3]Current addresss: 520 Olney Science Center, Department of Chemistry, Kennedy College of Science, University of Massachusetts Lowell, Lowell, MA 01854, USA. E-mail: suri_iyer@uml.edu.

Contents:

1. Apples-to-apples comparison of the assay described in the manuscript to a standard ELISA.
2. Characterization studies.

1. Apples-to-apples comparison of the assay described in the manuscript to a standard ELISA.

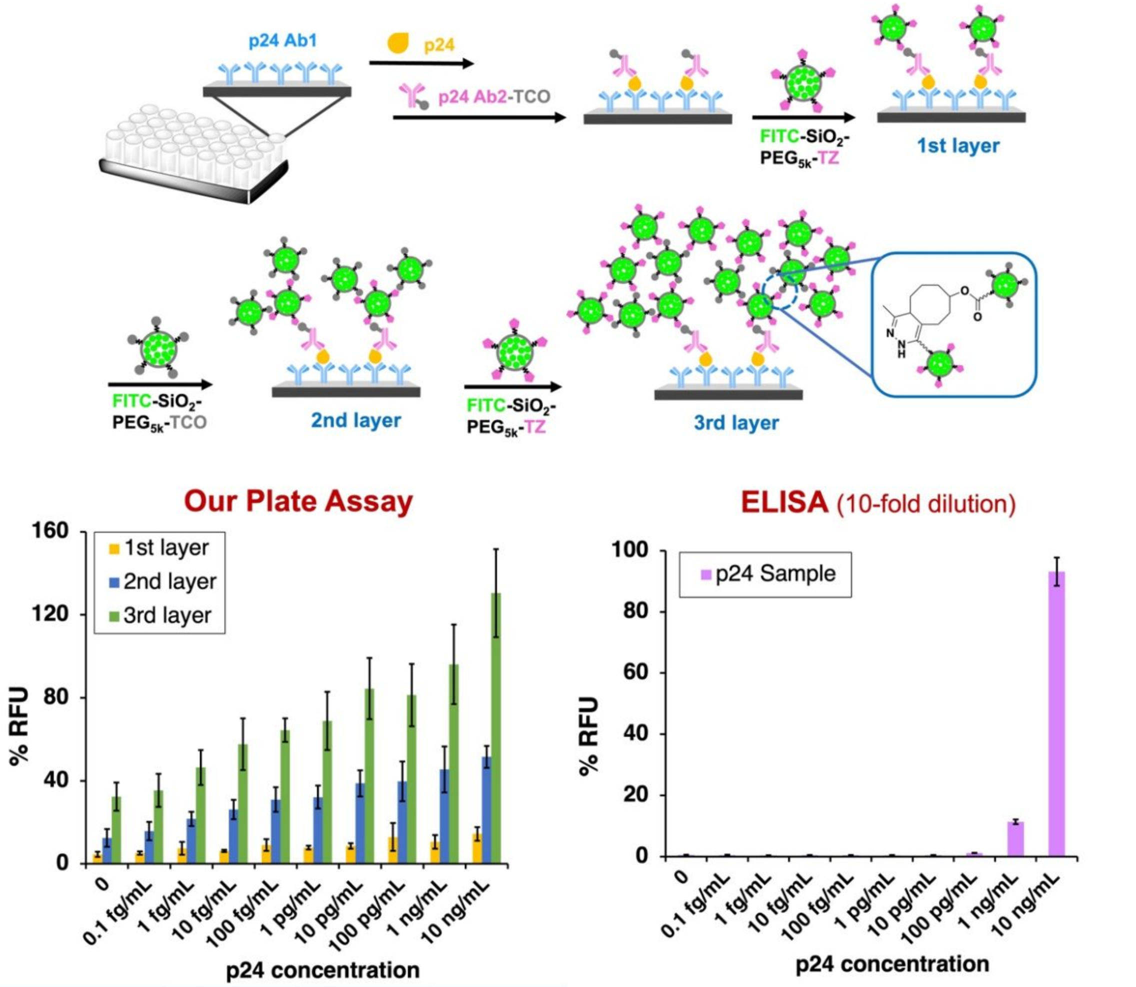

	1st layer	2nd layer	3rd layer
LOD (Plate assay)	209 fg/mL	10 fg/mL	8 fg/mL
LOD (Fe_3O_4)	100 pg/mL	100 fg/mL	17 fg/mL

- Linear detection range: 500 pg/mL – 8 pg/mL
- LOD: 1 pg/mL

Figure S1. *Apples to apples comparison to demonstrate the LOD and range of detection of our assay versus a standard commercially available ELISA assay for p24.* **Top:** Schematic of the microwell plate based assay. **Bottom:** Comparison between the two methods.

2. Characterization studies.

2.1. Transmission electron microscopy (TEM) for fluorescent silica nanoparticles

All samples were dispersed in ethanol and the materials were dropped onto a carbon-coated copper grid followed by evaporating the solvent at rt.

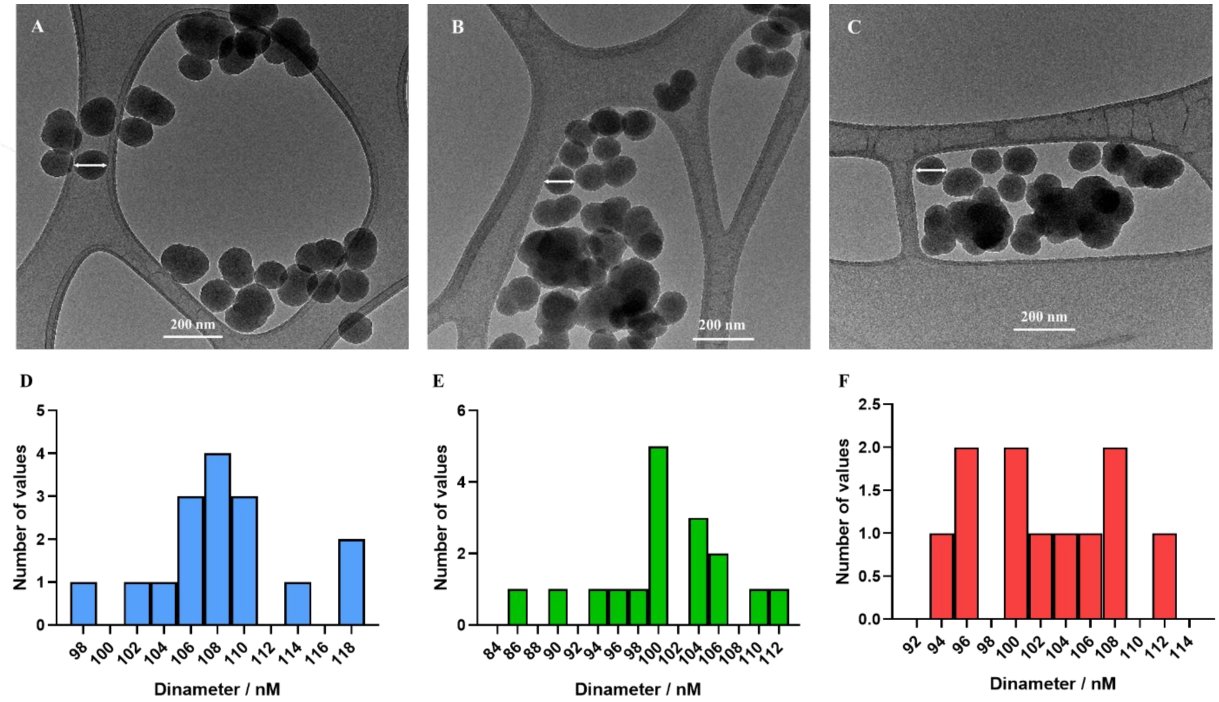

Figure S2. TEM images of fluorescent silica nanoparticles. (A) FITC-SiO_2-OH, (B) FITC-SiO_2-NH_2, (C) FITC-SiO_2-PEG5k-COOH. Size distribution of (D) FITC-SiO_2-PEG5k-OH (E) FITC-SiO_2-PEG5k-NH_2 and (F) FITC-SiO_2-PEG5k-COOH. (White arrows indicate the size of fluorescent silica nanoparticles).

2.2. Dynamic light scattering (DLS) measurement

All the samples were dispersed in a PBS buffer before measurement. DLS measured by Zetasizer Nano-zs90 from Malvern Instruments.

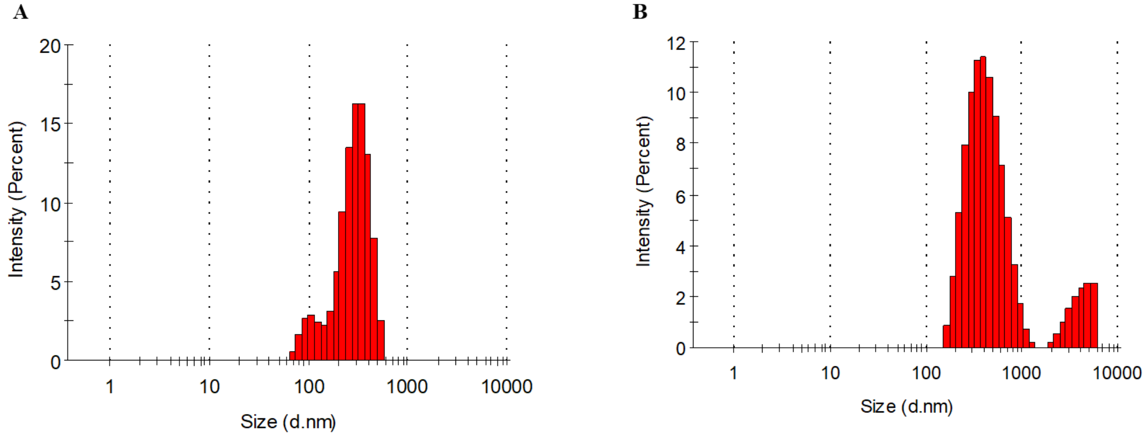

Figure S3. DLS measurement. (A) FITC-SiO_2-PEG5k-TCO. (B) FITC-SiO_2-PEG5k-TZ.

2.3. Zeta potential measurement

All the samples were dispersed in a PBS buffer before measurement. Zeta potential measured by Zetasizer Nano-zs90 from Malvern Instruments.

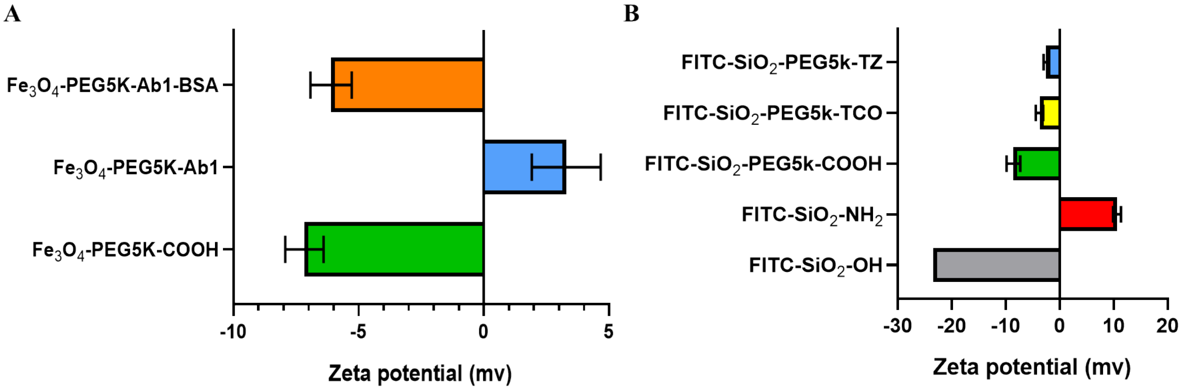

Figure S4. Zeta potential of magnetic nanoparticles (A) and fluorescent silica nanoparticles (B). Error bars indicate standard deviations of three measurements.

2.4. MALDI-TOF mass spectrometry for Tetrazine-modified antibody 2 (Ab2-TZ)

α-Cyano-4-hydroxycinnamic acid (Sigma Aldrich) was used as the MALDI matrix and was prepared by dissolving 2 mg in 0.1 mL 50/50 acetonitrile/water with 0.1% trifluoroacetic acid. The number of tetrazine molecules conjugated on the anti-HIV-1 antibody was determined by MALDI-TOF mass spectrometry based on changes in molecular weight. The 3500 Dalton change in molecular weight with the addition of the TZ corresponds to ~10 TZ molecules.

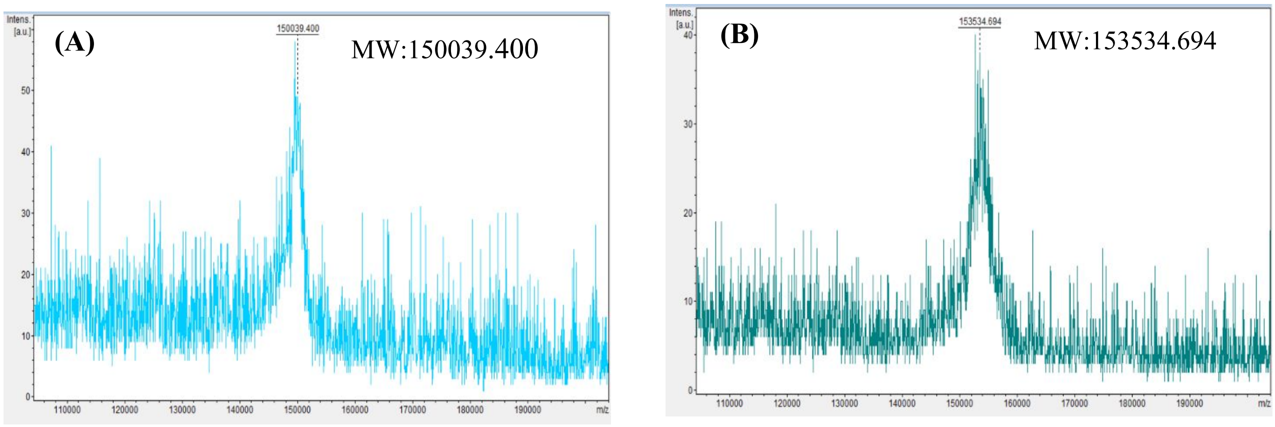

Figure S5. Mass spectrometry for Ab2-TZ. (A) unmodified anti-HIV-1 p24 antibody, (B) tetrazine-modified anti-HIV-1 p24 antibody.
